# Oxidation of Human Copper Chaperone Atox1 and Disulfide Bond Cleavage by Cisplatin and Glutathione

**DOI:** 10.3390/ijms20184390

**Published:** 2019-09-06

**Authors:** Maria I. Nardella, Antonio Rosato, Benny D. Belviso, Rocco Caliandro, Giovanni Natile, Fabio Arnesano

**Affiliations:** 1 Department of Chemistry, University of Bari, via Orabona, 4, 70125 Bari, Italy; 2 Institute of Crystallography, CNR, via Amendola, 122/o, 70126 Bari, Italy

**Keywords:** copper, cisplatin, Atox1, disulfide bond, metallochaperones, oxidative stress

## Abstract

Cancer cells cope with high oxidative stress levels, characterized by a shift toward the oxidized form (GSSG) of glutathione (GSH) in the redox couple GSSG/2GSH. Under these conditions, the cytosolic copper chaperone Atox1, which delivers Cu(I) to the secretory pathway, gets oxidized, i.e., a disulfide bond is formed between the cysteine residues of the Cu(I)-binding CxxC motif. Switching to the covalently-linked form, sulfur atoms are not able to bind the Cu(I) ion and Atox1 cannot play an antioxidant role. Atox1 has also been implicated in the resistance to platinum chemotherapy. In the presence of excess GSH, the anticancer drug cisplatin binds to Cu(I)-Atox1 but not to the reduced apoprotein. With the aim to investigate the interaction of cisplatin with the disulfide form of the protein, we performed a structural characterization in solution and in the solid state of oxidized human Atox1 and explored its ability to bind cisplatin under conditions mimicking an oxidizing environment. Cisplatin targets a methionine residue of oxidized Atox1; however, in the presence of GSH as reducing agent, the drug binds irreversibly to the protein with ammine ligands *trans* to Cys12 and Cys15. The results are discussed with reference to the available literature data and a mechanism is proposed connecting platinum drug processing to redox and copper homeostasis.

## 1. Introduction

Cancer cells have to cope with oxidative stress conditions, characterized by high levels of reactive oxygen species (ROS) [1]. Since many antineoplastic drugs used in chemotherapy contribute to increase the oxidative stress of target cells causing apoptosis [2], the ability to establish an imbalance between ROS and antioxidant levels may represent a useful strategy for cancer attack [3].

Platinum coordination complexes (such as cisplatin, carboplatin and oxaliplatin) and anthracyclines (such as doxorubicin, epirubicin and daunorubicin), used alone or in combination, generate extremely high ROS levels [2]. The widely used chemotherapy agent cis-diamminedichloridoplatinum(II) (best known as cisplatin), is employed for the treatment of a wide variety of solid malignancies and causes formation of DNA intra- and inter-strand cross-links [4,5], but only 1–4% of the drug is able to reach the nucleus [6,7,8,9,10]. Despite its large use, cisplatin treatment can often result in the development of resistance responsible for treatment failure [4,11,12].

Cells can develop cisplatin resistance by different mechanisms. It has been found that cell lines with high resistance to cisplatin have considerably higher levels of copper (Cu) chaperone Atox1 [13]. Atox1 chelates Cu(I) ions in the cytosol and delivers them to the Cu-transporting ATPases located in the trans-Golgi network, thus enabling the biosynthetic maturation of secreted Cu-dependent enzymes, such as ceruloplasmin [14,15,16]. Atox1 is a 68-amino acids protein featuring a ferredoxin-like fold (β_1_α_1_β_2_β_3_α_2_β_4_) and a metal-binding motif CxxC (located in the β_1_α_1_ loop) able to bind a single Cu(I) ion [17]. This structure is highly conserved among metallochaperones and soluble domains of Cu-ATPases [18]. It has been shown that Atox1 can also bind cisplatin in the cytosol and may prevent its transfer to the nucleus [19,20,21]. 

Neoplastic cells may survive oxidative stress by acquiring adaptive mechanisms to neutralize the potential toxic effects of elevated ROS [22,23,24]. These mechanisms of redox adaptation may involve different pathways, including the glutathione antioxidant system, the major ROS-scavenging system in the cells, capable of preventing damage caused by ROS to important cellular components [22,24]. Glutathione (GSH) is a tripeptide (Glu-Cys-Gly) with γ peptide linkage between the carboxyl group of a Glu side-chain and the amine group of Cys and a normal peptide linkage between the carboxyl group of Cys and the amine group of Gly. GSH, which is present in a concentration range of 0.5–10 mM in the cytosol, can react with hydrogen peroxide in a reaction catalyzed by glutathione peroxidase and can also react directly with ROS in non-enzyme-catalyzed reactions [25,26]. The resulting oxidized species (GSSG) contains an intermolecular disulfide bond between the Cys residues of two glutathione molecules and is reduced by NADPH-dependent glutathione reductase [25,26]. 

A decrease of the intracellular GSH/GSSG ratio, by GSH oxidation, switches the cell potential to more positive values, thus influencing the redox status of Atox1, which contains three Cys residues (Cys12 and Cys15, forming the CxxC motif, and Cys41, located far apart on strand β_3_), which can be found either in the reduced or in the oxidized form [27,28]. Thus, the GSSG/2GSH couple can modulate the redox state of the CxxC motif of Atox1 through a reaction catalyzed by human glutaredoxin 1 (Grx1), that promotes Atox1 oxidation by GSSG but not the complementary reduction of oxidized Atox1 by GSH, unless Cu(I) is present [29]. 

In this work, we investigated the effect of high oxidative stress conditions on Atox1 structure and ability to bind cisplatin. We found that cisplatin, in the presence of GSH, allows cleavage of the Atox1 S-S bond and binds to the disulfide-reduced form of the protein with ammine ligands *trans* to Cys12 and Cys15, respectively.

## 2. Results

### 2.1. Atox1 Oxidation

**Figure 1:**
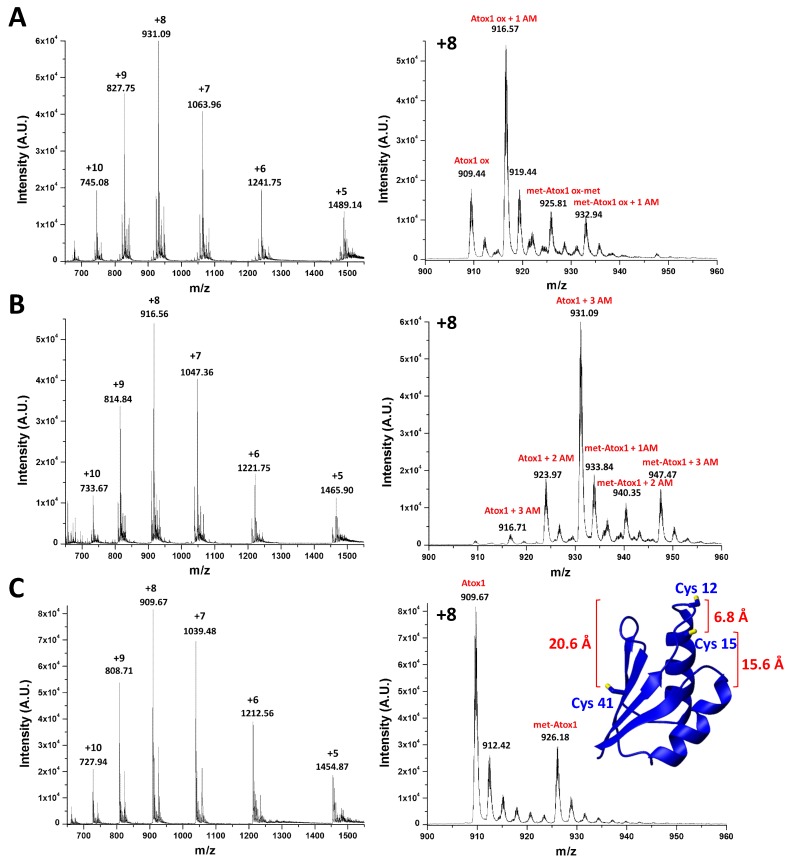
Electrospray mass spectrometry (ESI-MS) spectra of oxidized (**A**) and reduced (**B**) Atox1 treated with three equivalents of 2-iodoacetamide (IAM) and ESI-MS spectrum of the untreated reduced Atox1 (**C**). The spectra show two sets of peaks corresponding to the expected total masses with or without cleavage of the N-terminal methionine (met, 131 Da) of Atox1. (Inset) Average interatomic distances between sulfur atoms of cysteines residues of Atox1, displayed on the lowest energy structure of the reduced apoprotein (PDB ID 1tl5).

The complete oxidation of Atox1 was conducted by incubating the protein with three equivalents of GSSG for 12 h at 25 °C. Optimal reaction conditions for quantitative oxidation were chosen after several tests in order to avoid harsh treatments. To monitor the oxidation status of Atox1, electrospray mass spectrometry (ESI-MS) experiments were performed. Since the oxidized and reduced forms differ by only 2 Da, the analysis was carried out by incubating the protein in the dark for 30 min with 50 equivalents of 2-iodoacetamide (IAM) in 20 mM ammonium acetate (pH 6.3). IAM is a commonly used alkylating agent for free thiols in proteins [30]. Alkylation by IAM of reduced cysteines results in the covalent coupling of a carbamidomethyl (AM) group (57.1 Da). The ESI-MS spectra of reduced and oxidized Atox1 treated with IAM (Figure 1A,B), compared to those of the reduced, untreated protein (Figure 1C), show that the most abundant species in the first case corresponds to Atox1 + 3 AM and in the second case to Atox1 + 1 AM. This result indicates that two cysteines of oxidized Atox1 are involved in a disulfide bond, hence they are not subject to IAM modification. The average interatomic distances between sulfur atoms of Cys residues in *apo*Atox1 (mean values resulting from a family of thirty NMR structures, PDB ID 1tl5 [17], of 6.8 ± 1.0 Å, 20.6 ± 0.8 Å, and 15.6 ± 1.4 Å for Cys12–Cys15, Cys12–Cys41, and Cys15–Cys41, respectively) indicate that an *intramolecular* disulfide bond can be formed between Cys12 and Cys15 of the CxxC motif, while Cys41 can still be modified by IAM (Figure 1A).

### 2.2. Characterization of Oxidized Atox1 in Solution

In order to monitor the redox process by nuclear magnetic resonance (NMR) spectroscopy, three equivalents of GSSG were added to reduced, ^15^N-labeled *apo*Atox1 (70 μM), in the presence of EDTA (210 μM) to chelate any divalent metal cation impurity and of 10% *v/v* D_2_O for the field-frequency lock. The pH was fixed at 7.0 (with 25 mM phosphate buffer) and the temperature was kept constant at 298 K.

**Figure 2:**
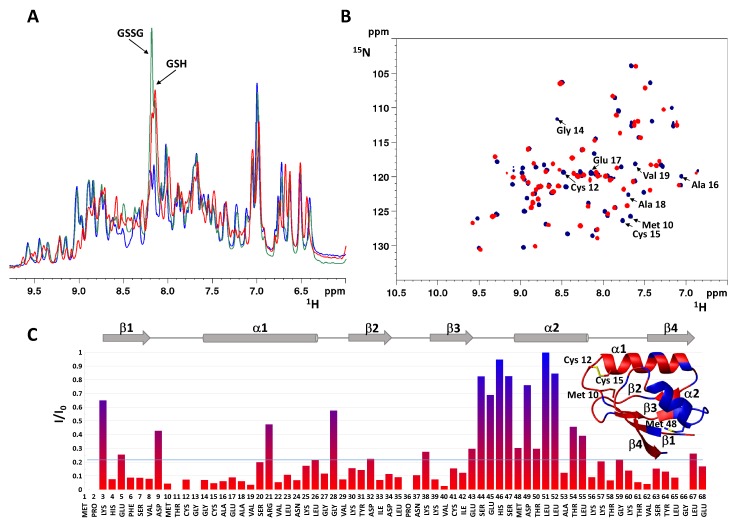
(**A**) Comparison of 1D ^1^H spectra of *apo*Atox1 in the reduced form (blue) and after 1 h (green) and 12 h (red) incubation with three equivalents of GSSG. The signals of the amide proton of the glycine of GSSG and GSH are indicated by arrows. (**B**) Overlay of 2D ^1^H,^15^N HSQC spectra of *apo*Atox1 in the reduced form (blue) and after 12 h incubation with GSSG (red). The arrows indicate cross-peaks of residues close to the CxxC motif for reduced *apo*Atox1. (**C**) Plot of intensity ratio (I/I_0_) versus the primary sequence, calculated at resonance positions of backbone amides of reduced *apo*Atox1, using ^1^H,^15^N HSQC spectra recorded after 12 h of incubation with GSSG (I) and in the absence of GSSG (I_0_). The secondary structure elements are shown on top. (Inset) Residues with I/I_0_ above average are mapped in blue on the structure of oxidized Atox1 and residues with I/I_0_ below average are mapped in red. The ribbon diagram was generated with the program MOLMOL.

**Figure 3:**
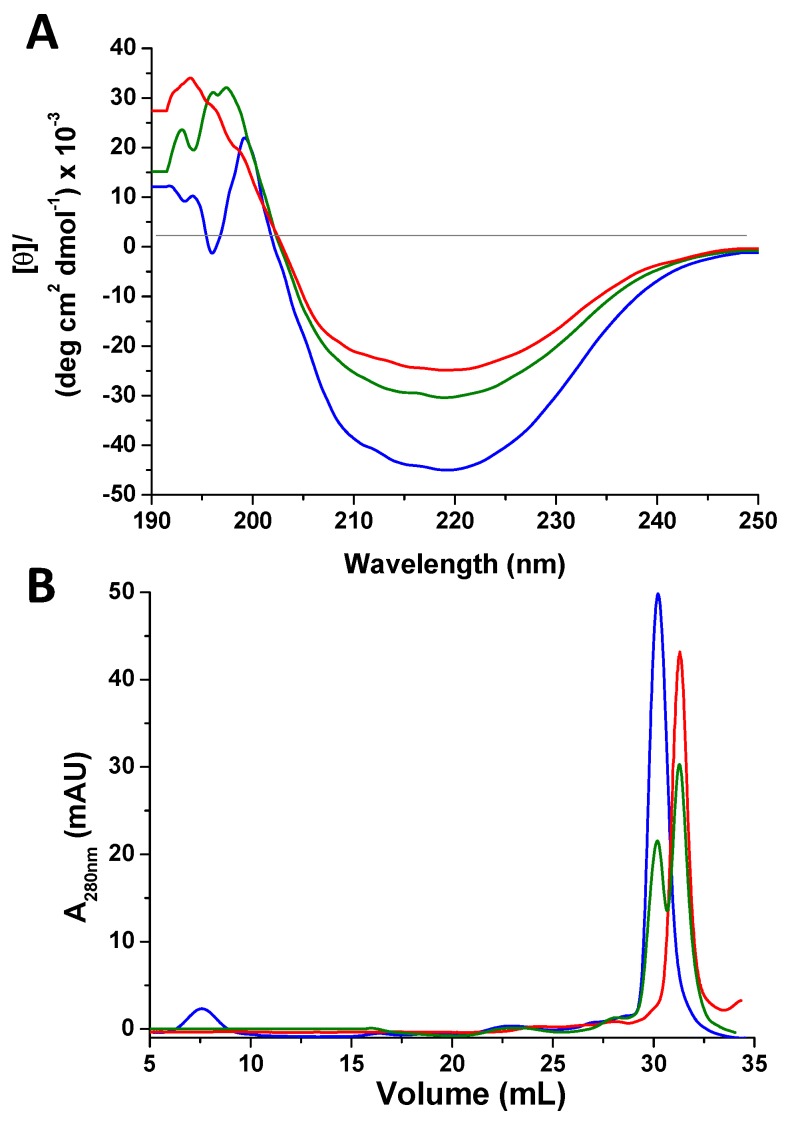
(**A**) Far-UV circular dichroism spectra in 25 mM phosphate buffer (pH 7.0) of *apo*Atox1 in the reduced form (blue) and after 1 h (green) and 12 h (red) incubation with three equivalents of GSSG, plotted as the mean residue molar ellipticity, [θ]. (**B**) Analysis by size-exclusion chromatography (SEC) of *apo*Atox1 in the reduced form (blue) and after 1 h (green) and 12 h (red) incubation with three equivalents of GSSG. The chromatography was carried out on a Superdex 75 Increase 10/300 column (GE Healthcare, column volume 24 mL) using 20 mM ammonium acetate (pH 6.5) at a flow rate of 0.5 mL min^−1^. The volume of protein solution injected was 0.5 mL for each run.

In 1D ^1^H NMR spectra recorded at different incubation times, the amide proton signal of the glycine of GSSG (at 8.2 ppm) decreases as a result of the redox process, while that of GSH (at 8.17 ppm) tends to increase (Figure 2A). The presence, throughout the reaction, of two sets of cross-peaks in the 2D ^1^H,^15^N heteronuclear single quantum coherence (HSQC) spectra for the amide groups of the protein indicates that the oxidized and the reduced form are in slow exchange on the NMR time scale (i.e., the interconversion rate is slower than milliseconds). After 12 h incubation with GSSG, the oxidation of Atox1 is complete and the HSQC spectrum shows several protein cross-peaks that experienced large variations of chemical shift with respect to the reduced form (Figure 2B). Spectral changes are not just localized in the protein region that encompasses the CxxC motif, but involve a larger portion of the protein, as shown by the intensity ratio diagram (Figure 2C), in which the less variable region corresponds to helix α_2_ (highlighted in blue in the inset). By observing the signal linewidths and the distribution of cross-peaks (Figure S1), it can be deduced that the oxidized form is a structured monomer, as also confirmed by circular dichroism (CD) and size-exclusion chromatography (SEC). The CD profile is similar for oxidized and reduced Atox1, apart from a decrease in ellipticity [θ] observed upon addition of GSSG to the solution (Figure 3A). The SEC behavior of oxidized Atox1 is slightly different from that of the reduced apoprotein and allows to monitor the oxidation process. The chromatograms (UV detector set at 280 nm) show two peaks eluting at a retention volume (RV) of 30.2 and 31.5 mL for the reduced and the oxidized protein, respectively (Figure 3B), consistent with monomeric size.

### 2.3. Preliminary Crystal Structure Characterization of Oxidized Atox1

Several Atox1 crystals were analyzed by X-ray diffraction. Main crystallographic parameters of the best dataset are reported in Table 1. Despite the low data resolution and huge data anisotropy, crystal cell parameters and crystal symmetry were promptly identified without ambiguities. The molecular replacement (MR) model (PDB ID 3iwl) was chosen based on similarity with crystal cell and packing, and in fact the MR solution in the target cell was found very close to the model in the original cell. The MR solution was then refined by using the coordinates of individual atoms as free variables. However, for most of the residues, the limited data resolution does not allow to determine univocally the side-chain conformation. Regarding the metal-binding site, the absence of any significant electron density peak in the region usually occupied by metal ions and the proximity of Cys12 and Cys15 lead us to conduct the refinement under the hypothesis of the presence of a disulfide bond between Cys12 and Cys15.

**Figure 4:**
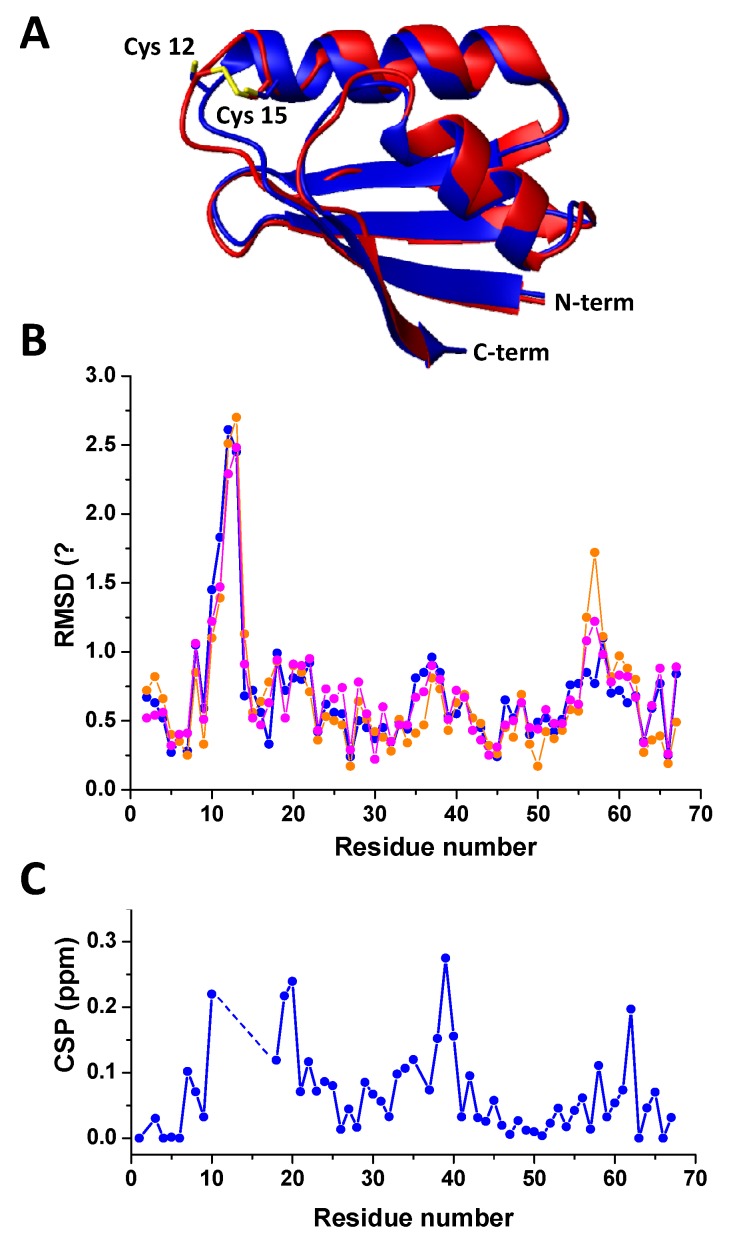
(**A**) Crystal structure of oxidized human Atox1 (red) overlaid on the structure of the monomeric human Atox1 with cisplatin (blue), used as molecular replacement model (PDB ID 3iwl). Sulfur atoms are shown in yellow. The cisplatin moiety is omitted for clarity. (**B**) Per-residue Cα root-mean-square deviation (RMSD) of oxidized human Atox1 with respect to monomeric human Atox1 with cisplatin (PDB ID 3iwl, blue line) and human Atox1 homodimer with oxaliplatin (4qot, orange line and magenta line for chains A and B, respectively). (**C**) Values of ^1^H,^15^N chemical shift perturbation (CSP) between oxidized and reduced Atox1, plotted for each assigned amino acid.

The final crystal structure (*R_work_*, 0.247, *R_free_* 0.268) is shown in Figure 4A. The distance between the S atoms of Cys12 and Cys15, which was 4.02 Å for the MR model, is 2.04 Å for oxidized Atox1. A clear electron density bump is present close to His46, in a region occupied by a water molecule or a sulfate ion in other Atox1 crystal structures, and it has been modeled with a water molecule. In Figure 4B, it is reported the residue-by-residue root-mean-square deviation (RMSD) between C_α_ atoms of oxidized Atox1 and other known human Atox1 crystal structures, namely that of the monomer (PDB ID 3iwl) [31] and of the homodimer (PDB ID 4qot) [32]. Main deviations, up to 2 Å, reside in the metal-binding region, comprised between Met10 and Cys15, where a Pt(II) ion is hosted in 3iwl and 4qot. Other residues having large deviations, which are above 1 Å for the homodimer, are Lys56 and Lys57, belonging to a lysine-rich region (KKTGK) located in the loop α_2_β_4_. This region is the nuclear localization signal for the translocation of Atox1 to the nucleus and plays a key role in the formation of the Atox1 dimer, representing a large portion of the intermolecular interaction surface. Notably, the plot of the RMSD values of the Atox1 crystal structures shows similarities with the backbone chemical shift perturbation (CSP) analysis (Figure 4C), pointing at similar conformational changes between oxidized and reduced Atox1 in solution and in the solid state.

### 2.4. Interaction with Cisplatin

The ^15^N chemical shifts of ammine ligands of cisplatin are extremely sensitive to the nature of donor atoms in *trans* position, therefore they can report changes in Pt coordination (the ^15^N nuclei resonate approximately between −30 and −50 ppm when trans to S, between −50 and −75 ppm when trans to Cl or N, and between −70 and −90 ppm when trans to O) [33].

**Figure 5:**
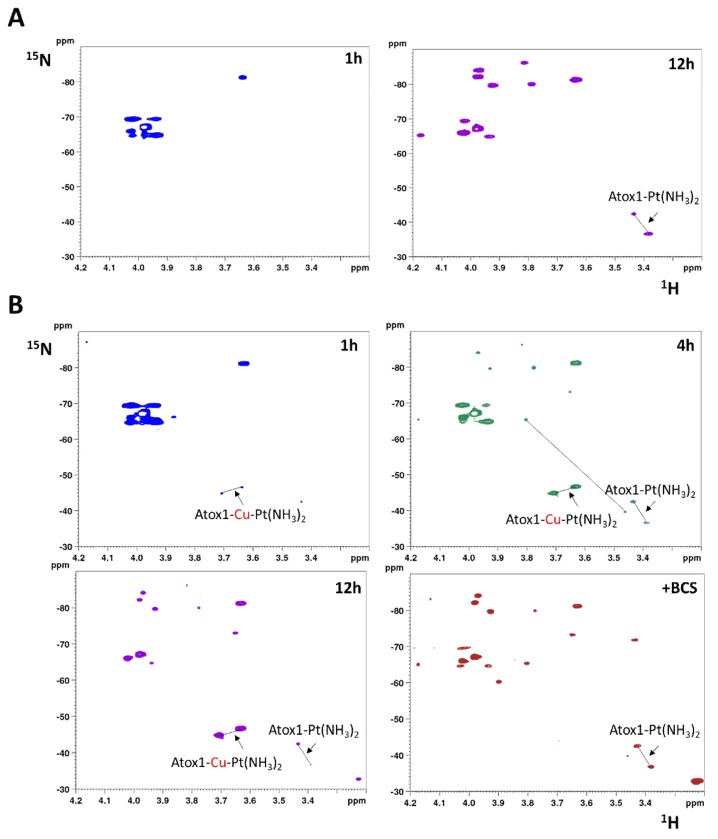
(**A**) Two-dimensional ^1^H,^15^N-HSQC spectra of ^15^N-labeled cisplatin recorded at two different time intervals after mixing with one equivalent of reduced *apo*Atox1 (70 μM) in 25 mM phosphate buffer (pH 7.0), in the presence of EDTA (210 μM). (**B**) Two-dimensional ^1^H,^15^N-HSQC spectra of ^15^N-labeled cisplatin recorded at different time intervals after mixing with one equivalent of Cu(I)-Atox1 (70 μM) in 25 mM phosphate buffer (pH 7.0), in the presence of GSH (210 μM) and EDTA (210 μM). After 12 h of incubation, bathocuproine disulfonate (BCS) was added.

When ^15^N-labeled cisplatin is incubated for 12 h with one equivalent of reduced *apo*Atox1 (70 μM), in addition to the cross-peaks of the dichloro (*cis*-[PtCl_2_(^15^NH_3_)_2_], δ ^15^N = 67 ppm) and the monochloro monoaqua species (*cis*-[PtCl(^15^(NH_3_)_2_(H_2_O)]^+^, δ ^15^N = −65 and −88 ppm for the ^15^NH_3_ ligand trans to Cl and trans to O, respectively), two cross-peaks appear in the ^1^H,^15^N-HSQC spectrum in the region of ^15^NH_3_ *trans* to S (δ ^15^N = −42.5 and −36.5 ppm), corresponding to the bidentate adduct Atox1-Pt(^15^NH_3_)_2_, with both cysteines of the CxxC motif of Atox1 replacing the chlorido ligands of cisplatin (Figure 5A) [34].

We previously demonstrated that *apo*Atox1 is unable to bind cisplatin in excess of GSH [34]. Remarkably, preloading with Cu(I) enhances the reactivity of Atox1 toward cisplatin notwithstanding the presence of GSH [34,35]. The reaction of ^15^N-labeled cisplatin with Cu(I)-Atox1 (70 μM) was carried out in the presence of 210 μM GSH. After 1 h incubation, two cross-peaks corresponding to ^15^N *trans* to S (δ ^15^N = −44.8 and −46.6 ppm) are detected (Figure 5B). After 4 h, in addition to these signals, which are downfield-shifted along the ^1^H dimension, the cross-peaks of Atox1-Pt(^15^NH_3_)_2_ [34] are observed along with those of the monodentate adduct of ^15^N-labeled cisplatin with reduced glutathione (δ ^15^N = −65.1 and −39.7 ppm, for ^15^NH_3_ ligand trans to Cl and trans to S, respectively) [36]. After 12 h, the cross-peaks of [PtCl(^15^NH_3_)_2_(GS)] disappear and those of Atox1-Pt(^15^NH_3_)_2_ become barely detectable, whereas the downfield signals show higher intensity. At this incubation time, addition of 210 µM bathocuproine disulfonate (BCS, a ligand which binds specifically Cu(I) with high affinity) [37] causes the immediate disappearance of the downfield signals and the increase of the Atox1-Pt(^15^NH_3_)_2_ cross-peaks, indicating that the downfield signals belong to a species of the type Atox1-Cu-Pt(^15^NH_3_)_2_ containing a Cu-Pt core (Figure 5B). These results indicate that Cu(I)-Atox1 favorably competes with GSH for binding to cisplatin.

**Figure 6:**
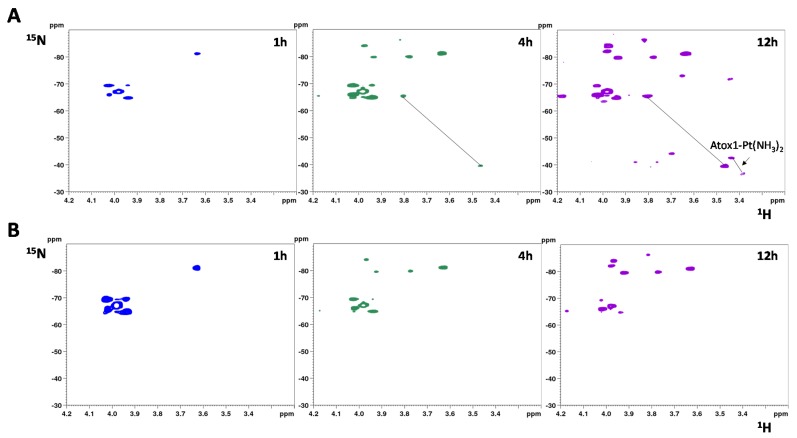
(**A**) Two-dimensional ^1^H,^15^N-HSQC spectra of ^15^N-labeled cisplatin recorded at different time intervals after mixing with one equivalent of oxidized Atox1 (70 μM) in 25 mM phosphate buffer (pH 7.0), in the presence of GSH/GSSG (140 μM/140 μM) and EDTA (210 μM). (**B**) Two-dimensional ^1^H,^15^N-HSQC spectra of ^15^N-labeled cisplatin recorded at different time intervals after mixing with one equivalent of oxidized Atox1 (70 μM) in 25 mM phosphate buffer (pH 7.0), in the presence of EDTA (210 μM) and in the absence of GSH/GSSG.

The reaction of ^15^N-labeled cisplatin with oxidized Atox1 (70 μM) was also monitored by NMR. The protein solution contained a mixture of GSSH and GSH (~1:1 molar ratio), as produced by the protein oxidation process. After 1 h incubation, only the dichloro and the monochloro monoaqua species are detected. After 4 h, in addition to the peaks generated by the hydrolysis of cisplatin, two new peaks of the monodentate adduct of cisplatin with GSH emerge and, only after 12 h of incubation, signals characteristic of Atox1-Pt(^15^NH_3_)_2_ are distinctly observed. Notably, these ^15^NH_3_ peaks resonate exactly at the same chemical shift of those observed in the reaction with reduced *apo*Atox1 in the absence of GSH (δ ^15^N/^1^H = −42.5/3.43 and −36.5/3.39 ppm), indicating that cisplatin binds to the reduced protein after cleavage of the disulfide bond between the two cysteines of the CxxC motif (Figure 6A).

It can be hypothesized that the reduction takes place at the expense of GSH, in analogy with what has been observed in the case of Cu(I) [29]. Indeed, monitoring the reaction between ^15^N-labeled cisplatin and oxidized Atox1 in the absence of GSH/GSSG (removed from the reaction solution with Amicon filters having a cutoff of 3 kDa), even after 12 h of incubation no signals of Atox1-Pt adducts were observed (Figure 6B). If, however, GSH is added at the end of the incubation period, the platinum adduct with Atox1 starts to form, confirming the essential role of GSH.

**Figure 7:**
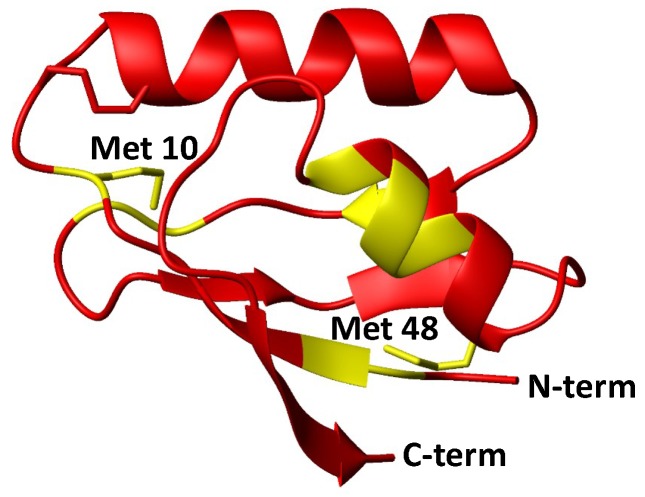
Mapping of residues with an intensity ratio (I/I_0_) below average, shown in yellow on the structure of oxidized Atox1 given in red. The I/I_0_ ratio was calculated at resonance positions of backbone amides of oxidized Atox1, using ^1^H,^15^N HSQC spectra recorded in the presence of GSH/GSSG after 1 h of incubation with cisplatin (I) and in the absence of cisplatin (I_0_).

The same reaction was monitored looking at the changes in the HSQC spectrum of the protein. The ratio of cross-peak intensities before and after addition of cisplatin to oxidized Atox1 in the presence of GSH/GSSG highlights a region of the protein encompassing residues in the proximity of Met48 (Figure 7). Notably, this is the region least affected by the formation of the intramolecular disulfide bond (Figure 2C, *inset*). Also residues in close contact with Met10 are affected by cisplatin, but at a lesser extent (Figure 7)

## 3. Discussion

ESI-MS and NMR experiments show that a slight excess of GSSG (3:1, GSSG:protein ratio) quantitatively oxidizes human Atox1 forming an intramolecular disulfide bond between the cysteine residues of the CxxC motif. Upon oxidation, the protein remains soluble and well-folded in a monomeric state, but with a rather different HSQC spectrum, indicating that structural changes propagate beyond the metal-binding site. A preliminary X-ray structure characterization shows that the crystal packing is similar to that exhibited by the only other known crystal structure of human Atox1 monomer, i.e., the reduced protein bound to Pt (PDB ID 3iwl, and its revision 4ydx). The limited data resolution (3.9 Å) achieved does not allow to unambiguously model the side-chain conformations for all residues, and in particular to directly assess the presence of a disulfide bond between Cys12 and Cys15. However, the backbone conformation of the metal-binding site with an S-S crosslink has been generated and validated, showing compatible agreement with the X-ray data. While the overall fold is conserved, the structural model differs from all other known Atox1 crystal structures mainly for a different conformation of loop α_2_β_4_, with Lys56 and Lys57 showing the highest local RMSD values. It is not clear whether this change in conformation is, at least in part, induced by crystallization (an agarose hydrogel was used to drive the crystallization process, which had never been used before for Atox1 studies), or is a consequence of the different conformation of the dithiol motif. In support of this latter hypothesis, the CSP analysis shows that the NMR spectral changes following protein oxidation are localized in analogous regions of the protein as in the solid state. Further crystallization trials are ongoing and are showing that crystallization still occurs in the absence of agarose, although the crystal growth is significantly slowed down (months vs. days).

Solution NMR data indicate that the disulfide bond in Atox1 remains intact after addition of cisplatin and in the absence of added GSH. Instead, a slow cleavage of the disulfide bond occurs when both GSH and cisplatin are present in the solution, with Pt(II) stabilizing the reduced form by forming the bidentate species Atox1-Pt(NH_3_)_2_ [34,35].

It is known that intra- and intermolecular disulfide bonds can be broken in the presence of platinum complexes [38,39,40,41,42]. Possible mechanisms are:

1. Direct attack of Pt on the disulfide (RSSR acting as monodentate ligand), followed by cleavage of the disulfide bond through formation of sulfinic acid (RSO_2_H) [39]. In at least one case, it was proposed that a divalent metal ion, such as Ni(II), can bind to a protein disulfide in the absence of thiol reduction [43], but in the case of Atox1 there is no evidence of formation of Pt(II) adduct with oxidized cysteines which would have produced ^15^NH_3_ chemical shifts different from those of the adduct of cisplatin with reduced *apo*Atox1. Instead, all minor peaks in HSQC spectra belong to cisplatin hydrolyzed forms or adducts with GSH that are removed by filtration using a 3 kDa cutoff.
2. Initial hydrolysis of disulfide bond to give thiol and sulfenic acid (RSSR + H_2_O → RSH + RSOH) followed by cisplatin coordination to one thiol (as proposed for Ag^+^) [44], but in the case of Atox1 there is no binding of cisplatin to thiol(s) in the absence of GSH/GSSG. In a condition where no binding of ^15^N-labeled cisplatin is detected, the perturbation of intensity of ^1^H,^15^N protein cross-peaks can be informative about changes in the chemical environment at the protein surface owing to weak interactions with the drug. The result of this analysis indicates that cisplatin perturbs the protein region around Met48, which is far from the CxxC motif and is not affected by disulfide formation. Slight variations in intensity are also detected at the Met10 site, involving residues in close contact with its buried side-chain [45]. The poor accessibility of Met10 would hamper the binding of cisplatin; however, a conformational change induced by disulfide formation may project the side-chain of Met10 into the solvent, similarly to what occurs with the homologous residue Met12 in the oxidized form of the mercury-binding protein MerP [46]. Analogously to Atox1, cisplatin does not bind to cysteines in fully oxidized Cox17 (the copper chaperone for cytochrome *c* oxidase) and results indicate that a methionine residue could be the binding site [47].
3. Involvement of glutathione in the reaction mechanism. In the present case, the monofunctional adduct [PtCl(NH_3_)_2_GS] is formed in the presence of GSH/GSSG, which could also mediate the interaction with oxidized Atox1. The NMR spectra did not show intermediate species that could encompass the protein and the Pt moiety (e.g., monofunctional adducts of cisplatin with the protein), therefore Pt(II) *chelate* binding appears to be very efficient once a good template structure is obtained. In this regard, the reaction of Cu(I)-Atox1 with cisplatin is indicative, since it is found that when GSH is present in slight excess, the bidentate adduct contains a Cu(I) ion, located in the vicinity of Pt(II), whose role is to keep the two cysteines very close to one another, thus allowing the chelate binding of cisplatin (and possibly of other *cis* compounds). When a disulfide bond is present between the two cysteines, the S-S distance is lower compared to Cu(I)-Atox1 (2.0–2.1 vs. 3.6–3.7 Å) and Pt binding is only possible provided that a source of reducing equivalents is found nearby. It might be argued that some residual reduced apoprotein present in the GSH/GSSG solution can bind cisplatin instead of oxidized Atox1. However, it has been shown that reduced *apo*Atox1 is not able to bind cisplatin in the presence of excess GSH [34], thus it is inferred that formation of Atox1-Pt(NH_3_)_2_ in GSH/GSSG should occur through a *concerted* mechanism that involves the oxidized protein and GSH. Pt-thioether type adducts (especially with Met10) may serve as a drug reservoir for subsequent delivery to CxxC once the disulfide is reduced.

**Scheme 1:**
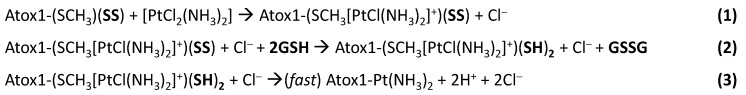
Possible reaction mechanism of oxidized Atox1 with cisplatin and cleavage of disulfide bond in the presence of reduced glutathione (GSH).

In our proposed mechanism, the Pt-containing species present in solution (i.e., intact cisplatin and its hydrolyzed forms) first interact with a methionine residue of oxidized Atox1, and when reduction is accomplished by GSH, Pt(II) binding stabilizes the reduced form, thus increasing the redox potential and shifting the reaction to the right (Scheme 1).

## 4. Materials and Methods

### 4.1. Preparation of GSH and Cisplatin Solutions

^15^N-labeled cisplatin, prepared by literature method [48], was dissolved prior to use in ultrapure deoxygenated water at 1 mg/mL final concentration. The Pt complex solution was extensively vortexed and sonicated for 10 min at 25 °C. GSSG (Sigma-Aldrich, St. Louis, MO, USA) was dissolved in ultrapure deoxygenated water at final concentration of 11 mM. All samples were manipulated in anaerobic conditions (N_2_ atmosphere) using a glovebox (ItecoEng, Castelbolognese, Italy).

### 4.2. Protein Expression and Purification

Unlabeled and uniformly labeled ^15^N-Atox1 were recombinantly expressed by biotechnological techniques, using E. coli BL21DE3-Gold cells (Stratagene, La Jolla, CA, USA) and pET vectors under the control of the IPTG (Isopropyl β-d-1-thiogalactopyranoside) inducible lac promoter (Novagen, Madison, WI, USA). Purification was achieved by combining cation-exchange and size-exclusion chromatography. In particular, Atox1 expression was induced at 37 °C with 1 mM of IPTG (Sigma-Aldrich) for 4 h. All purifications steps were carried out in the presence of an excess of the reducing agent dithiothreitol (DTT, Sigma-Aldrich) in order to preserve the reduced form of the protein. An automated AKTA-Purifier UPC900, equipped with a detector at 280 nm and proprietary columns (all from GE Healthcare, Amersham, UK), was used in all cases. Protein purity was determined by sodium dodecyl sulfate polyacrylamide gel electrophoresis (SDS-PAGE), ESI-MS, and NMR. To extensively remove DTT prior to reactions, the apoprotein samples were extensively washed with 25 mM sodium phosphate buffer at pH 7.0, by using Amicon Ultra centrifugal filters with 3 kDa cutoff (Millipore, Burlington, MA, USA). The loading of Cu was carried out in the anaerobic chamber by adding to the protein solution an equimolar amount of tetrakis(acetonitrile)copper(I) hexafluorophosphate ([Cu(CH_3_CN)_4_]PF_6_) freshly prepared immediately prior to use. To obtain the oxidized protein, reduced apoAtox1 (70 μM) in phosphate buffer (25 mM, pH 7.0) was incubated at 25 °C for 12 h with three equivalents of GSSG under N_2_ atmosphere.

### 4.3. Size-Exclusion Chromatography

The progress of the oxidation reaction was monitored by SEC analysis of aliquotes (500 μL) of the incubation mixture taken at different time intervals. Gel filtration was performed with a Superdex 75 Increase 10/300 column (GE Healthcare, column volume 24 mL) on an AKTA purifier. The run was performed by using 20 mM ammonium acetate (pH 6.5) as running buffer, with a flow rate of 0.5 mL min^−1^, and monitoring the protein elution by measuring absorbance at 280 nm. The protein fractions corresponding to peaks of reduced and oxidized Atox1 were individually collected, concentrated with Amicon Ultra centrifugal filters (3 kDa cutoff), and analyzed by mass spectrometry.

### 4.4. Electrospray Mass Spectrometry

Electrospray mass spectrometry was performed with a 6530 Series Accurate-Mass Quadruple Time-of-Flight LC/MS (Agilent, Santa Clara, CA, USA). The samples were injected at a rate of 10 L/min. 1% Acetic acid (*v*/*v*) was added before injection to obtain a good volatilization. Ionization was achieved in the positive ion mode by application of 3.5 kV at the entrance of the capillary; the pressure of the nebulizer gas was 20 psi. The drying gas was heated to 325 °C at a flow rate of 10 L/min. Full-scan mass spectra were recorded in the mass/charge (*m*/*z*) range of 50–3000.

### 4.5. Nuclear Magnetic Resonance Spectroscopy

The oxidation of the protein and its reaction with ^15^N-labeled cisplatin were monitored through ^1^H,^15^N spectra, acquired using a gradient-enhanced sequence in which coherence selection and water suppression were achieved via gradient pulses. Sixteen transients were acquired over an F2 (^1^H) spectral width of 14 ppm into 2048 complex data points for each of 256 t1 increments with an F1 spectral width of 45 ppm centered at 115 ppm (for amide ^15^N) or 100 ppm centered at −50 ppm (for ^15^NH_3_). The sequence was optimized with a delay 1/(4J_NH_) of 2.78 ms and a recycle delay of 1.5 s.

All spectra were collected using an Avance 700 MHz UltraShield Plus magnet (Bruker Biospin, Rheinstetten, Germany), equipped with Quadruple Resonance (QCI) CryoProbe, and processed using the standard Bruker software (TOPSPIN). The backbone chemical shift perturbations (CSP) between oxidized and reduced Atox1 were calculated using the formula CSP = {[(δ_N_/5)^2^ + (δ_H_)^2^]/2}^1/2^, where δ_N_ and δ_H_ represent respectively the changes in ^15^N and ^1^H chemical shifts upon disulfide bond formation [49].

### 4.6. Circular Dichroism

Circular dichroism (CD) experiments were performed on a Jasco J-810 spectropolarimeter (Jasco Inc., Easton, MD, USA) at 25 °C. Quartz cuvettes with path length of 0.1 cm were used in the far-UV region from 190 to 250 nm. Each spectrum, obtained by averaging of five scans, was baseline-corrected and then smoothed by applying adjacent averaging or a fast Fourier transform filter. The ellipticity is reported as mean residue molar ellipticity (deg cm^2^ dmol^−1^) according to [θ] = 100 [θ]_obs_/(*C L N*), where [θ]_obs_ is the observed ellipticity in degrees, *C* is the molar concentration of the protein, *L* is the optical path length (in cm), and *N* is the number of amino acids (*N* = 68 for Atox1). Spectra were collected using reduced and oxidized Atox1 samples (obtained upon incubation of the protein with GSSG) in phosphate buffer (25 mM, pH 7.0) at 25 °C. The concentration of both samples was 10 μM.

### 4.7. Protein Crystallization

Oxidized Atox1 was produced in phosphate buffer (25 mM, pH 7) by adding GSSG (1:3, Atox1:GSSG molar ratio). Before setting up crystallization experiments, the protein has been concentrated to 12.4 mg/mL. Crystallization experiments were performed in nitrogen-filled glovebox to minimize the presence of oxygen in the crystallization wells. Three days of equilibration at 20 °C of 5 μL drop made of 2 μL of protein solution, 2 μL of reservoir solution, and 1 μL of agarose low strength 1% *w*/*v* (HR8-092, Hampton Research) against reservoir solution containing (NH_4_)_2_SO_4_ 2.8M, MES 0.1M at pH 6.0, and glycerol 3% *v*/*v*, allowed to obtain needle crystals of about 10 μm in size.

### 4.8. X-ray Diffraction Data Collection and Analysis

Atox1 crystals were analyzed by using the X-ray beam supplied at Diamond Light Source of Oxford, beamline I03. The presence of glycerol in the mother liquor in which the crystals had grown ensured good cryoprotection and minimized the presence of ice rings in the diffraction images. Data collections were carried out in cryogenic conditions (100 K) and at beam energy of 12,700 eV. The X-ray detector software (XDS) package [50] was used to perform data reduction, while POINTLESS and AIMLESS programs [51] were used to find space group symmetry and to scale and merge diffraction data. Data showed severe anisotropy, which was corrected by using the UCLA Diffraction Anisotropy Server [52]. Among the 2332 reflections in the initial set, 820 were discarded because they fell outside the ellipsoid defined by the recommended resolution limits of 4.9 Å, 4.9 Å and 3.8 Å along a *, b *, and c *, respectively. The remaining 1512 reflections were then anisotropically scaled. The anisotropy correction lowered the resolution limit from 3.79 to 3.9 Å. The crystal structure was solved by molecular replacement (MR), by using the REMO program [53] included in the package SIR2014 [54], and the crystal structure with PDB ID 3iwl [31] as MR model. The MR model was chosen after an extensive analysis, where all available crystal structures of human Atox1 (single chain) were tried independently as input for MR, and as best model was chosen that producing the best MR figure of merits. Structural refinement was performed by using phenix.refine [55], included in the PHENIX package [56]. Least-square twin refinement was carried out, by using the twin law (-h, -k, l) obtained from phenix.xtriage [57], which was detected with a multivariate Z score of 7.732 and a twin fraction (H alpha) of 0.148. Between automatic refinements, structural models were manually inspected and validated by using the program COOT [58].

## 5. Conclusions

Under physiological conditions (0.5–10 mM GSH) [59], cisplatin can hardly bind to reduced Atox1, unless Cu is present. When Pt binds to the cysteines of Cu(I)-Atox1, the Cu(I) ion is labilized and can be released into the cytosol, possibly causing oxidative stress. Under oxidizing conditions (low GSH/GSSG ratio), Atox1 switches to the disulfide form and Pt binds to the cysteines only in the presence of GSH as a source of reducing equivalents; this prevents further binding of Cu to Atox1 and its detoxification. Although the CxxC motif of oxidized Atox1 does not react with cisplatin (or the reaction is very slow), the reduction of disulfide in vivo can be catalyzed by Grx1 as it occurs for Cu [27,28,29]. Atox1 is able to undergo reversible reduction/oxidation under normal conditions; however, upon Pt binding and irreversible thiol blockage, Cu buffering (and export) can be compromised as well as redox homeostasis.

Increased ROS production is often associated with chemotherapy-induced apoptosis and is an early event in tumor responses to treatment [22,23,24]. Depletion of GSH increases the tumor-specific cytotoxicity of several chemotherapeutic drugs without increasing toxicity to normal tissues; in contrast, the ability of some cancer cells to maintain a highly reducing intracellular environment has been correlated with tumor aggressiveness and drug resistance [26,60,61]. On the other hand, the co-administration of Pt drug and a Cu chelator, such as tetrathiomolybdate, can increase the nuclear availability of cisplatin and resensitize resistant cells [62,63,64]. Notably, depletion of Cu and GSH disfavors the irreversible binding of the drug to reduced and oxidized Atox1, respectively, thus counteracting the role of the Cu chaperone in cisplatin resistance.

## Figures and Tables

**Table 1:** Crystallographic parameters obtained for datasets with and without anisotropy correction. Values in parentheses are for the highest resolution shell.

	Without Anisotropy Correction	With Anisotropy Correction
Resolution range (Å)	45.95–3.79 (4.24–3.79)	45.95–3.90 (4.36–3.90)
Space group	P3_2_21	P3_2_21
Crystal Cell (Å) *a*,*c*	53.06, 56.48	53.06, 56.48
Total reflections	2332 (627)	1512 (156)
Unique reflections	954 (245)	606 (50)
Completeness (%)	93.8 (91.2)	62.7 (17.0)
Multiplicity	2.4 (2.6)	2.5 (3.1)
<I/σ>	4.8 (1.8)	6.6 (5.5)
R_merge_ (%)	9.9 (43.8)	8.1 (14.4)
R_meas_ (%)	12.2 (53.2)	10.0 (17.3)
CC_1/2_	0.995 (0.904)	0.994 (0.996)
Mosaicity (°)	0.312	0.312
B Wilson (Å^2^)	74.4	50.1
R_work_	0.305	0.247
R_free_	0.343	0.268
Number of water molecules	1	1

## Supplementary Materials

The following are available online at https://www.mdpi.com/1422-0067/20/18/4390/s1.

## Abbreviations

AM	Carbamidomethyl
Atox1	Antioxidant 1 Copper Chaperone
BCS	Bathocuproine Disulfonate
CD	Circular Dichroism
CSP	Chemical Shift Perturbation
EDTA	Ethylenediaminetetraacetic Acid
ESI-MS	Electrospray Mass Spectrometry
Grx1	Glutaredoxin 1
GSH	Reduced Glutathione
GSSG	Oxidized Glutathione dimer
NADPH	Reduced Nicotinamide Adenine Dinucleotide Phosphate
HSQC	Heteronuclear Single Quantum Coherence
IAM	2-Iodoacetamide
MR	Molecular Replacement
NADPH	Reduced Nicotinamide Adenine Dinucleotide Phosphate
NMR	Nuclear Magnetic Resonance
PDB	Protein Data Bank
RMSD	Root-Mean-Square Deviation
ROS	Reactive Oxygen Species
RV	Retention Volume
SEC	Size-Exclusion Chromatography
