# Supplementary material for: Oxidation of Human Copper Chaperone Atox1 and Disulfide Bond Cleavage by Cisplatin and Glutathione
Source: Int J Mol Sci. 2019 Sep 6;20(18):4390. doi: 10.3390/ijms20184390 (PMC6769983; doi:10.3390/ijms20184390)
Supplement: Supplementary file 1 [file ijms-20-04390-s001.pdf]

SUPPLEMENTARY INFORMATION

Oxidation of human copper chaperone Atox1 and disulfide bond cleavage by cisplatin and glutathione

Maria I. Nardella [1], Antonio Rosato [1], Benny D. Belviso [2], Rocco Caliandro [2], Giovanni Natile [1] and Fabio Arnesano [1]

[1] Department of Chemistry, University of Bari, via Orabona, 4, 70125 Bari, Italy
[2] Institute of Crystallography, CNR, via Amendola, 122/o, 70126 Bari, Italy

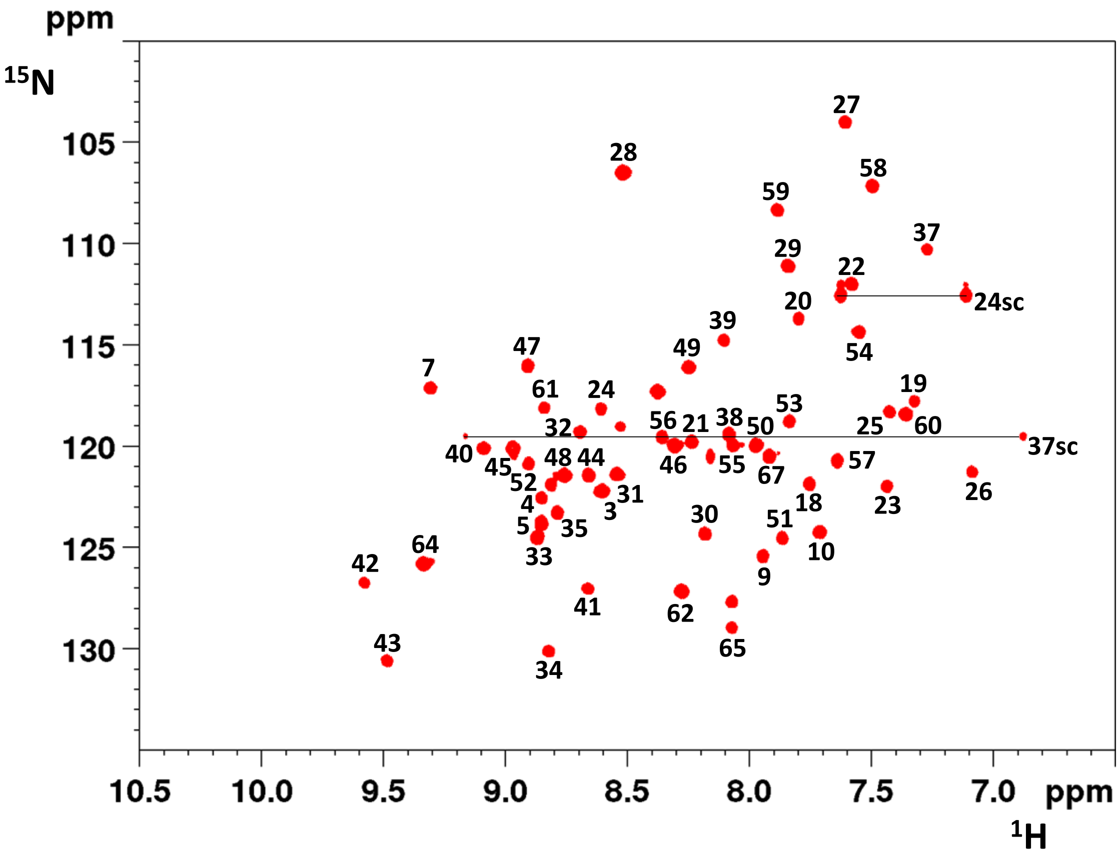

Supplementary Figure S1. 2D ^{1}H,^{15}N HSQC spectrum of oxidized Atox1 (red). The assigned cross-peaks are indicated.
